# Growth factor independence 1 ameliorates osteoarthritis by inhibiting chondrocyte ferroptosis via inactivation of MAPK signaling pathway

**DOI:** 10.1016/j.jot.2025.07.003

**Published:** 2025-07-29

**Authors:** Xiaoyu Jin, Xunhao Wang, Siyu Xu, Nuo Xu, Ziwei Wang, Chunqing Hu, Wei Liu, Zhaofeng Zhang, Xiyu Liu, Jingjing Fan, Ruiyang Jiang, Rui Wu, Zhongyang Lv, Dongquan Shi

**Affiliations:** a Division of Sports Medicine and Adult Reconstructive Surgery, Department of Orthopedic Surgery, Nanjing Drum Tower Hospital Clinical College of Nanjing University of Chinese Medicine, 321 Zhongshan Road, Nanjing 210008, Jiangsu, China; b Division of Sports Medicine and Adult Reconstructive Surgery, Department of Orthopedic Surgery, Nanjing Drum Tower Hospital, Affiliated Hospital of Medical School, Nanjing University, 321 Zhongshan Road, Nanjing 210008, Jiangsu, China; c Department of Orthopedics, Nanjing Jinling Hospital, Affiliated Hospital of Medical School, Nanjing University, Nanjing 210002, Jiangsu, China; d Suzhou Medical College of Soochow University, Suzhou 215031, Jiangsu, China; e Division of Sports Medicine and Adult Reconstructive Surgery, Department of Orthopedic Surgery, Nanjing Drum Tower Hospital Clinical College of Nanjing Medical University, 321 Zhongshan Road, Nanjing 210008, Jiangsu, China

**Keywords:** Osteoarthritis, Ferroptosis, Chondrocyte, Gfi1, MAPK

## Abstract

**Background:**

Osteoarthritis (OA) is the most common degenerative joint disease, characterized by cartilage deterioration, which is closely associated with chondrocyte ferroptosis. The aim of this study was to investigate the role and mechanism of previously unexplored gene, growth factor independence 1 (*Gfi1*) in chondrocyte ferroptosis, in order to provide a new therapeutic target for OA.

**Methods:**

The expression of ferroptotic hallmarks and Gfi1 were analyzed in human and mice OA cartilages and tert-butyl hydroperoxide (TBHP)-induced primary chondrocytes. Small interfering RNA or overexpression plasmids were used to knock down or overexpress *Gfi1* to explore its role in chondrocyte ferroptosis and metabolism. Then, the role of Gfi1 in destabilization of medial meniscus (DMM) surgery-induced mice OA model was investigated with or without the intra-articular injection of adeno-associated virus-overexpressing *Gfi1* (AAV-*Gfi1*). Furthermore, RNA sequencing analysis was performed to reveal the key downstream pathway of Gfi1 exerting its role in chondrocyte ferroptosis.

**Results:**

The expression of Gfi1 was significantly decreased, while 4-HNE, a typical lipid peroxidation product, was significantly increased both in damaged human and DMM surgery-induced mice OA cartilages. Consistently, Gfi1 was remarkably downregulated in TBHP-induced ferroptotic chondrocytes. Moreover, *Gfi1* knockdown aggravated chondrocyte ferroptosis by elevated levels of ferroptotic hallmarks, including total ROS, lipid ROS and Fe^2+^ accumulation. The upregulation of ferroptotic driver (Cox2, Acsl4) and catabolic marker (Mmp13) and downregulation of ferroptotic suppressors (Gpx4, Fth1, Slc7a11) and anabolic marker (Col II) were also observed in TBHP-induced chondrocytes by *Gfi1* knockdown. On the contrary, *Gfi1* overexpression showed anti-ferroptotic effect in TBHP-induced chondrocytes. Intra-articular injection of AAV-*Gfi1* evidently alleviated cartilage degeneration by resisting ferroptosis and preserving the anabolism-catabolism homeostasis in OA cartilages. Comprehensive evaluation of subchondral bone sclerosis, osteophyte formation, synovitis and behavior performance further validated that *Gfi1* overexpression ameliorated OA progression. Mechanistically, MAPK signaling pathway was identified as the key downstream mediator of Gfi1 exerting anti-ferroptotic role in OA.

**Conclusion:**

Gfi1 is downregulated in OA and its overexpression ameliorates OA progression by inhibiting chondrocyte ferroptosis via inactivation of MAPK signaling pathway.

**The translational potential of this article:**

This study identifies Gfi1 as a novel therapeutic anti-ferroptotic target for cartilage degeneration, providing more clues for optimizing OA treatment strategies in clinical practice.

## Graphical abstract

**Figure:**
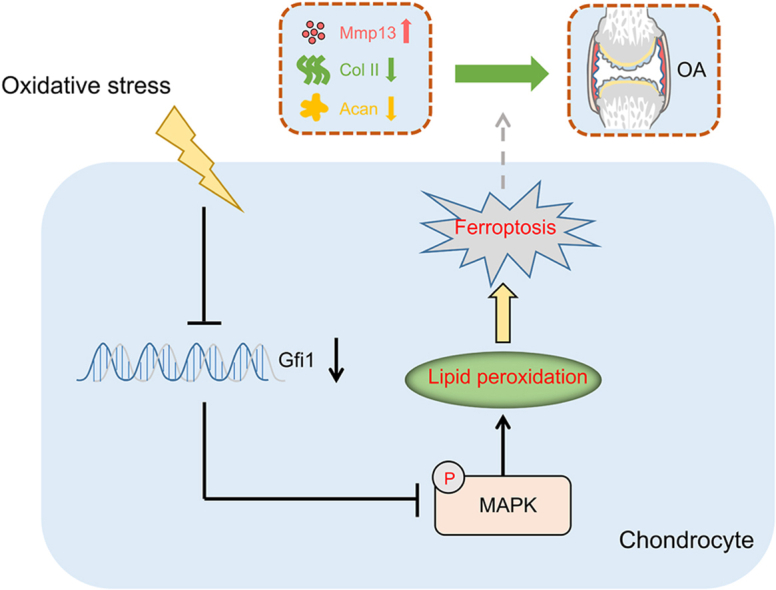

## 1 Introduction

Osteoarthritis (OA) is reckoned as the most frequent degenerative whole joint disease, symptomatically displayed as pain and dysfunction and pathologically characterized by cartilage degeneration, synovitis and abnormal subchondral bone remodeling [1,2]. The prevalence of OA exhibits raised trends over the past 30 years and affects an estimated 7.6 % of global population with the predicted upregulation of 60–100 % by 2050 [3], which implies the escalated socioeconomic burden towards the whole world. Nevertheless, there's still lack of effective disease-modifying strategy for OA due to unclarified pathogenesis. Therefore, it's essential to explore underlying mechanisms and identify potential therapeutic targets for OA.

Cartilage degeneration is the primary pathogenesis of OA, within which chondrocyte ferroptosis has been reported to be involved [4,5]. Distinguished from other types of cell death, like pyroptosis, apoptosis, and necroptosis, ferroptosis which was firstly proposed by Dixon et al., in 2012, exhibits unique features in morphology, genetics, biochemics and immunology [[6], [7], [8]]. As a neoteric programmed cell death mode, ferroptosis is initiated by disrupted redox homeostasis due to overloading iron-induced ROS production by Fenton reaction and featured with excessive iron load and cumulative lipid peroxidation [6,9]. Although chondrocyte ferroptosis has been substantially verified to contribute to OA cartilage degeneration [[10], [11], [12]], effective anti-ferroptotic targets in chondrocytes remain scarce.

Growth factor independence 1 (Gfi1) is a DNA binding zinc finger protein, which primarily acts as a repressive modulator in cancer and hemopathy [[13], [14], [15]]. It was reported that Gfi1 transcriptionally suppressed SOCS1 expression in acute myeloid leukemia cells and promoted the proliferation and migration of esophageal squamous cell carcinoma cells through restraining SOCS1 [14,15]. Moreover, Gfi1 was also shown to reduce the activity of tumor suppressor P53 via recruiting its LSD1 structure to silence target gene [16,17]. These studies imply the anti-ferroptotic potential of Gfi1, as demonstrated by its inhibitory impacts on ferroptotic drivers (e.g., SOCS1, P53) [[18], [19], [20]]. It's worth noting that a recent study has provided convincing evidence that Gfi1 could directly regulate osteoblast ferroptosis by inhibiting bone marrow mesenchymal stem cells-derived exosomal miR-150-3p in steroid-induced osteonecrosis of the femoral head [21]. However, whether Gfi1 protects chondrocytes from ferroptosis is still unknown.

In this study, we observed the downregulation of Gfi1 both in OA cartilages and tert-butyl hydroperoxide (TBHP)-induced ferroptotic chondrocytes, and elucidated the dual role (sensitize/protect) of *Gfi1* knockdown or overexpression in chondrocyte ferroptosis. Additionally, intra-articular injection of AAV-*Gfi1* evidently mitigated chondrocyte ferroptosis, cartilage degeneration and OA progression. Furthermore, MAPK signaling pathway was revealed as the downstream mediator of Gfi1 exerting anti-ferroptotic role in OA chondrocytes by RNA sequencing and experiment validation. Overall, this work may provide a potential therapeutic target for anti-ferroptosis-based OA therapy.

## 2 Materials and methods

### 2.1 Clinical specimen

The articular cartilage specimens from knee OA patients (60–84 years old, Kellgren–Lawrence grade 4) undergoing total knee arthroplasty were collected and divided into intact (lateral condyle) and damaged (medial condyle) areas as previously reported [11]. Among them, 4 pairs were used for protein extraction and western blot analysis and another 6 pairs were utilized for histological analysis. The clinical characteristics of OA patients were exhibited in Supplementary Tables 1–2. The study protocol was approved by the Ethical Committee of Nanjing Drum Tower Hospital, the Affiliated Hospital of Nanjing University (2022-176-02). All patients had signed informed consent before their participation.

### 2.2 Animal experiment

All animal experiment procedures were approved by the Animal Care and Use Committee of Nanjing Drum Tower Hospital, the Affiliated Hospital of Nanjing University (DWSY-24168626). Wild type C57BL/6 male mice were purchased from Model Animal Research Center of Nanjing University (Jiangsu, China) and kept in specific pathogen-free (SPF) condition. Destabilization of medial meniscus (DMM) surgery was conducted on the right knees of 12-week-old C57BL/6 mice to establish an OA model. The operation procedures of Sham groups were performed as DMM surgery, except destabilizing meniscus. Experimental subjects were randomly divided into 4 groups (n = 6 per group): (i) Sham + AAV-NC group; (ii) DMM + AAV-NC group; (iii) Sham + AAV-*Gfi1* group; (iv) DMM + AAV-*Gfi1* group. Referring to manufacturer's instructions, adeno-associated virus-negative control (AAV-NC) or adeno-associated virus-overexpressing *Gfi1* (AAV-*Gfi1*) (PackGene, China) was injected into mice intra-articular cavity 3 weeks before surgery, so as to guarantee the transfection efficacy. Eight weeks after surgery, all experimental mice were sacrificed and their right knee joint samples were harvested and fixed in 4 % paraformaldehyde (PFA) solution for further analysis.

### 2.3 Cell culture

The primary mouse chondrocytes were extracted and cultured according to previous protocols [22]. Chondrocytes were cultured in Dulbecco's modified Eagle's medium (Gibco, USA) with the addition of 10 % fetal bovine serum (Gibco, USA) and 1 % penicillin and streptomycin (Gibco, USA) at 5 % CO_2_ and 37 °C condition. The cell culture medium was replaced each 48h, and chondrocytes from 1 to 2 generations were applied in cell experiments. Chondrocytes were treated with 100 μM TBHP (#MKCH9944, Sigma, USA), 10 μM MAPK-IN-1 (#2470587-69-8, MCE, China), small interfering RNA (siRNA) or overexpression plasmid of *Gfi1* (Zebrafish Biotech, China).

### *2.4* *Gfi1* knockdown and overexpression in chondrocytes

Scramble siRNA (Scr) and si-*Gfi1* were purchased from Zebrafish Biotech (Nanjing, China). The qualified siRNA of G*fi1* used in this study was synthesized as the following sequences: 5′-GCAAGAAGGCGCACAGCUATT-3’. The transfection procedure of siRNA was basically conducted as previously described [23]. Zebrafish Biotech (Nanjing, China) also provided us with qualified *Gfi1*-overexpression plasmid and negative control plasmid. Primary mouse chondrocytes subjected to the plasmid transfection with the addition of Opti-MEM (#31985070, Gibco, USA), Lipofectamine 3000 (#L3000015, Thermo Fisher Scientific, USA) and P3000 (#L3000015, Thermo Fisher Scientific, USA), according to the manufacturer's instructions. Following cell experiments were performed at 24 or 48 h after completing transfection.

### 2.5 Protein extraction and western blot analysis

The total protein from human cartilages and primary mouse chondrocytes were both extracted by utilizing RIPA lysis buffer (#R0010, Solarbio, China) including 1 mM phosphatase inhibitor cocktail (#B15002, Bimake, USA) and 1 mM phenylmethanesulfonyl fluoride (#329-98-6, Solarbio, China). The protein concentrations were measured by utilizing BCA Protein Assay Kit (#23225, Thermo Fisher Scientific, USA). Western blot experiment was performed referring to our previous procedures [23]. The primary antibodies applied in western blot were as follows: anti-COL2A1 (#BA0533, Boster, China), anti-MMP13 (#GB11247-1-100, Servicebio, China), anti-COX2 (#GB11077-1-100, Servicebio, China), anti-GFI1 (#14198-1-AP, proteintech, China), anti-GPX4 (#ab125066, Abcam, UK), anti-FTH1 (#4393, Cell Signaling Technology, USA), anti-ACSL4 (#A20414, Abclonal, China), anti-SLC7A11 (#DF12509, Affinity, USA), anti-Erk1/2 (#4695, Cell Signaling Technology, USA), anti-p-Erk1/2 (#5726, Cell Signaling Technology, USA), anti-SAPK/JNK (#9252, Cell Signaling Technology, USA), anti-p-SAPK/JNK (#4668, Cell Signaling Technology, USA), anti-P38 MAPK (#8690, Cell Signaling Technology, USA), anti-p-P38 MAPK (#AP0526, abclonal, China) and anti-β-Actin (#4970S, Cell Signaling Technology, USA). After incubation with secondary antibody-a horseradish peroxidase-conjugated goat anti-rabbit/mouse IgG (#BL003A or #BL001A, Biosharp, China), SuperKine™ West Femto Maximum Sensitivity Substrate (#BMU102, Abbkine, China) was used for western blot imaging in the ChemiDocXRS + Imaging System (Tanon, Shanghai, China). ImageJ software (version 1.8.0, USA) was adopted for the quantitative analysis of target proteins.

### 2.6 Quantitative real-time polymerase chain reaction (qPCR) analysis

The mRNA extraction from primary mouse chondrocytes with various treatment was conducted by RNA-Quick Purification Kit ((#RN001, ES Science, China) in accordance with manufacturer's instructions. The complementary DNA (cDNA) was obtained from mRNA after utilizing reverse transcription reagents (#R323-01, Vazyme, China). qPCR experiment was performed by amplifying 20 μL of diluted cDNA with ChamQ Blue Universal SYBR qPCR Master Mix (#Q312-02, Vazyme, China) and relevant primers on a LightCycler 480 PCR System (Roche, Switzerland). All primer sequences applied in this study were listed in Supplementary Table 3.

### 2.7 Cell live/dead staining and cell viability detection

The cells were inoculated on 12/96-well plates with specific treatment according to the experimental design. After multiple washing by phosphate buffer saline (PBS), AM/PI staining (#C2015S, Beyotime, China) or CCK8 kit (#CK04, Dojindo, Japan) was utilized to respectively assess the live/dead state or cell viability of treated chondrocytes as manufacturer's instructions described.

### 2.8 Measurement of intracellular Fe^2+^, ROS and lipid ROS levels

Fe^2+^ probe FerroOrange (#F374, Dojindo, Japan), ROS probe DCFH-DA (#S0033S, beyotime, China) and lipid ROS probe C11-BODIPY (#GC40165, GLPBIO, USA) were utilized for the measurement of intracellular Fe^2+^, ROS and lipid peroxidation levels by co-incubation with chondrocytes for 30 min at 5 % CO_2_ and 37 °C condition. After washing the probes with PBS, primary mouse chondrocytes were induced by 100 μM TBHP for 5 h with or without pretreated siRNA or overexpression plasmid of *Gfi1*. The probe stainings were observed and captured by a fluorescence microscope (Zeiss, Germany) and quantified by ImageJ software (version 1.8.0, USA). Intracellular fluorescent intensity of above probes was measured by BD AccuriC6 Plus Flow CytoMeter (BD Biosciences, USA) and analyzed by FlowJo software (Version 10.8.1, USA).

### 2.9 Safranin O-fast green (S.O.), hematoxylin & eosin (HE) and tissue ROS staining

The harvested samples of human cartilages and mice knee joints were fixed in 4 % PFA for 48 h, and subsequently decalcified in 10 % EDTA solution (#1340, Biofroxx, Germany). After completing decalcification, samples were embedded in paraffin and cut into continuous coronal 5 μm thick slides by a microtome (Thermo Fisher Scientific, USA). The slides were selected for Safranin-O/fast green (S.O.) (#G1371, Solarbio, China) and HE (#C0105S, Beyotime, China) staining to assess the degree of cartilage degeneration and synovitis, respectively according to the Osteoarthritis Research Society International (OARSI) (0–6) grading and synovitis (0–3) scoring systems by double-blinded observers [24]. The ROS levels in mice knee cartilages were evaluated by tissue ROS staining (#BB-460522, Bestbio, China) according to the manufacturer's instructions.

### 2.10 Immunohistochemical and immunofluorescence staining

The primary mouse chondrocytes were fixed in 4 % PFA for 30 min and subsequently permeated with 0.3 % Triton X-100 for 20 min. The deparaffinized histological slides of human cartilage or mice knee joints underwent antigen repair treated with 0.25 % Trypsin–EDTA (Gibco, USA) for 1 h at 37 °C. After multiple PBS washing, pretreated chondrocytes or slides were blocked by 5 % bovine serum albumin (BSA) for 1 h at room temperature and then were incubated overnight (4 °C) with primary antibodies as follows: anti-COL2A1 (#BA0533, Boster, China), anti-Aggrecan (#13880-1-AP, proteintech, China), anti-MMP13 (#GB11247-1-100, Servicebio, China), anti-GFI1 (#14198-1-AP, proteintech, China), anti-4-HNE (#ab46545, Abcam, UK), anti-COX2 (#GB11077-1-100, Servicebio, China), anti-GPX4 (#ab125066, Abcam, UK), and anti-FTH1 (#4393, Cell Signaling Technology, USA). After multiple washing, the chondrocytes or slides were incubated with a fluorescein isothiocyanate (FITC)-coupled secondary antibody (Thermo Fisher Scientific, USA) for 1 h at room temperature. Then, cell nucleus was stained with DAPI (#AB104139, Abcam, UK). Representive images in immunofluorescence (IF) staining were observed and captured with a fluorescence microscope (Zeiss, Germany). For immunohistochemical (IHC) staining, 3 % (v/v) H_2_O_2_ was additionally utilized to quench the activity of endogenous peroxidase before antigen repair. A horseradish peroxidase-conjugated goat anti-rabbit immunoglobulin G (IgG) (#BL003A, Biosharp, China) was used as the secondary antibody (1 h, room temperature). Visualization of IHC staining was performed by an ultra-sensitive DAB Kit (#1205250, Typing, Nanjing, China). Nonimmune IgG was utilized as the negative control both in IF and IHC staining. Further quantitative analysis of IF and IHC stainings were conducted by double-blinded investigators.

### 2.11 Micro-computed tomography (micro-CT) analysis

Before decalcification, mice knee joints with various treatment were scanned via a micro-CT scanner (mCT80, Scanco Medical AG, Switzerland), and 3D reconstruction of mice knees was performed via Scanco Medical software (Scanco threshold: 220). Then, we calculated the number of osteophytes by observing the 3D reconstructive images of knee joints and analyzed the subchondral bone parameters including: subchondral plate thickness (SBP), bone mineral density (BMD), the ratio of bone volume to tissue volume (BV/TV), trabecular number (Tb.N), trabecular thickness (Tb.Th) and trabecular separation (Tb.Sp).

### 2.12 Behavior experiments

Before sacrifice, the experimental mice were put in an open field at the size of 50 cm length × 50 cm width under the silent and semi-dark environment. Mice spontaneous moving trajectory was subsequently recorded for 3 min by a tracking system (Zhenghua Technology, China), and their activity parameters (relative activity, active time, distance and mean speed) were comprehensively evaluated. For gait analysis, the experimental mice hind paws were dipped with red ink and front paws were dipped with blue ink. Then, the footprints were recorded at a 20 cm × 70 cm self-made walking orbit with the bottom covered by white paper and mice step, stride and front/rear print were further assessed. The pain sensitivity was evaluated by mice hind paw withdrawal mechanical threshold measuring via an electronic von Frey Anesthesiometer (IITC, Woodland Hills, USA). All data were measured and analyzed by double-blinded observers.

### 2.13 RNA sequencing analysis

Total RNAs were isolated from primary mouse chondrocytes induced by 100 μM TBHP for 5 h with or without pretreated *Gfi1* overexpression (n = 3). Acquired RNAs were submitted to GeneChem company (Shanghai, China) for RNA sequencing. Differentially expressed genes (DEGs) were identified as fold change >1 and padj <0.05. DEGs were presented as volcano plots and heatmaps. Gene Ontology (GO), Kyoto Encyclopedia of Genes and Genomes (KEGG) and Gene Set Enrichment Analysis (GSEA) were conducted for the evaluation of DEGs’ biological function.

### 2.14 Statistical analysis

Experimental data were presented as the mean ± standard deviation (SD), and statistically analyzed and graphed by GraphPad Prism software (version 8.0, USA). The quantitative results were obtained by at least 3 independent repeated experiments. Shapiro–Wilk and Brown–Forsythe test were adopted to assess the data normal distribution and homogeneity of variance. Welch's test was further used to correct the statistical data with inhomogeneous variances. Paired or unpaired two-tailed Student's t test was utilized to compare 2 experimental groups. For comparing the data among more than 2 groups, we utilized one-way analysis of variance (ANOVA) followed by Tukey's post-hoc test. Statistical differences were considered significant when P < 0.05.

## 3 Results

### 3.1 Gfi1 is downregulated in OA cartilage and TBHP-induced ferroptotic chondrocytes

**Fig. 1:**
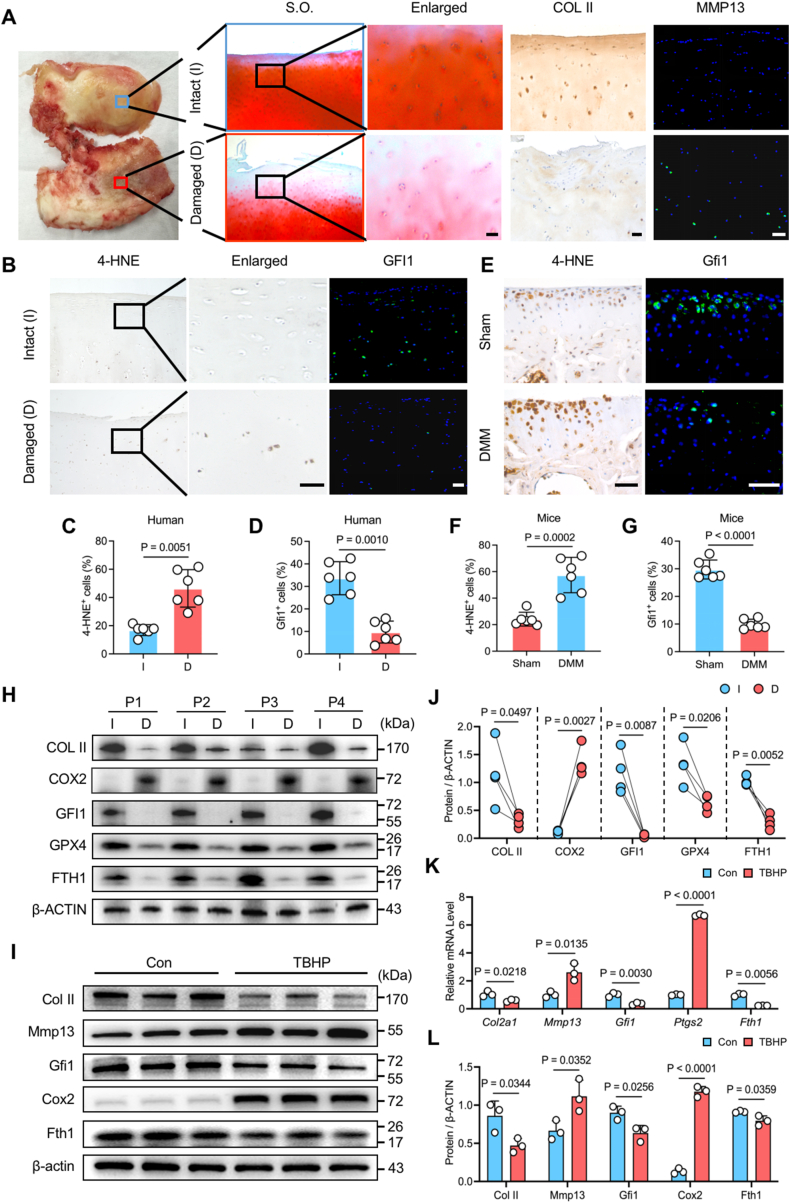
**Gfi1 is downregulated in OA cartilage and TBHP-induced ferroptotic chondrocytes. (A)** Specimen diagram, Safranin O-Fast Green (S.O) staining, Immunohistochemical (IHC) staining of COL II and immunofluorescence (IF) staining of MMP13 in intact (I) and damaged (D) human OA cartilages. **(B**–**D)** IHC staining of 4-HNE, IF staining of GFI1 **(B)** and their quantitative analysis **(C, D)** in intact (I) and damaged (D) human OA cartilages (n = 6). **(E**–**G)** IHC staining of 4-HNE, IF staining of Gfi1 **(E)** and their quantitative analysis (**F, G**) in the cartilages of Sham or destabilization of medial meniscus (DMM) surgery-induced OA mice (n = 6). **(H, J)** Western blot analysis **(H)** and quantification **(J)** of indicated proteins in paired intact (I) and damaged (D) human OA cartilages (n = 4). **(K)** qPCR analysis of indicated genes in primary mouse chondrocytes treated with 100 μM TBHP for 5 h (n = 3). **(I, L)** Western blot analysis **(I)** and quantification **(L)** of indicated proteins in primary mouse chondrocytes treated with 100 μM TBHP for 5 h (n = 3). P, patient. Scale bars, 50 μm. Two-tailed paired **(C, D, J)** or unpaired **(F, G, K, L)** t-test. Data are presented as mean ± SD.

To explore whether Gfi1 took part in chondrocyte ferroptosis, we investigated the expression of Gfi1 in cartilages both from OA patients and DMM surgery-induced OA mice. The damaged human OA cartilages were defined from morphology and histology, characterized by cartilage erosion shown in S.O. staining and extracellular matrix (ECM) disruption shown as increased Mmp13^+^ chondrocytes and reduced Col II^+^ areas in IF and IHC staining (Fig. 1A). Notably, the proportion of Gfi1^+^ cells were decreased in damaged cartilages, within which positive cells of 4-HNE, a primary lipid peroxidation product, accounted increasingly, indicating the existence of ferroptosis [11] (Fig. 1B–D). The similar trend was observed in mice OA model (Fig. 1E–G). At the protein level, the reduced expression of Col II, indicator of cartilage anabolism, also distinguished the damaged human OA cartilages (Fig. 1H and J). Meanwhile, the protein expression of Gfi1 was also significantly downregulated in damaged cartilages, accompanied by ferroptosis occurring with the increased ferroptotic driver, including cyclooxygenase 2 (Cox2), and decreased ferroptotic suppressors, including glutathione peroxidase 4 (Gpx4) and ferritin heavy chain 1 (Fth1) (Fig. 1H and J). Furthermore, as previously reported [11,25,26], we selected TBHP to effectively induce chondrocyte ferroptosis, as evidenced by increased expression of Cox2 and reduced expression of Fth1 (Fig. 1I, K and L). Simultaneously, the ECM metabolism homeostasis in chondrocytes was evidently disturbed by elevated levels of Mmp13 and declined levels of Col II (Fig. 1I, K and L). Notably, the mRNA and protein level of Gfi1 were both remarkbly downregulated in TBHP-induced ferroptotic chondrocytes (Fig. 1I, K and L). These findings indicate that Gfi1 expression is downregulated in ferroptotic chondrocytes both in vitro and vivo.

### 3.2 RNA sequencing analysis reveals the anti-ferroptotic potential of Gfi1 in chondrocytes

**Fig. 2:**
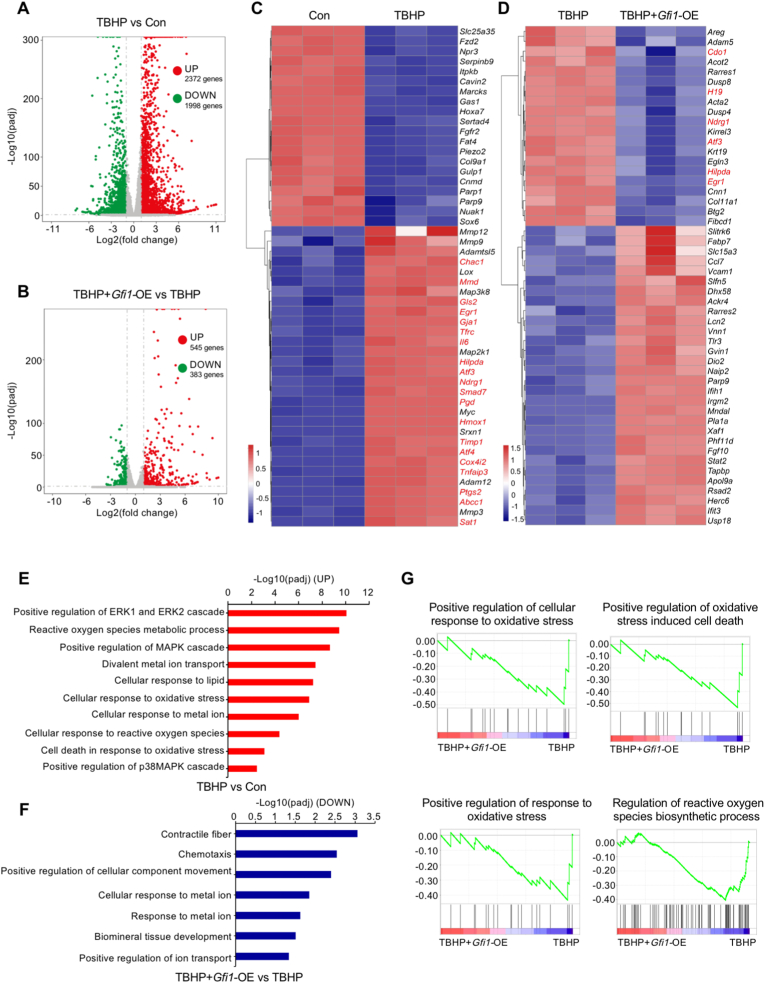
**RNA sequencing analysis reveals the anti-ferroptotic potential of Gfi1 in chondrocytes. (A, B)** RNA sequencing volcano plots between TBHP vs Con groups **(A)** and TBHP + *Gfi1*-OE vs TBHP groups **(B)**. **(C, D)** Heatmaps of differentially expressed genes (DEGs) between TBHP vs Con groups **(C)** and TBHP + *Gfi1*-OE vs TBHP groups **(D)** (n = 3). **(E)** Gene Ontology (GO) analysis of significantly upregulated genes between TBHP vs Con groups. **(F)** GO analysis of significantly downregulated genes between TBHP + *Gfi1*-OE vs TBHP groups. **(G)** Gene Set Enrichment Analysis (GSEA) in RNA sequencing.

In order to further elucidate the functional role of Gfi1 in chondrocyte ferroptosis, we performed RNA sequencing analysis on primary mouse chondrocytes induced by TBHP in the presence or absence of *Gfi1* overexpression. As shown in the volcano plots, 4370 DEGs containing 2372 upregulated and 1998 downregulated genes were identified between TBHP vs Con groups, while 928 DEGs containing 545 upregulated and 383 downregulated genes were identified between TBHP + *Gfi1*-OE vs TBHP groups (Fig. 2A and B). The heatmaps of DEGs illustrated that the expression levels of ferroptotic drivers, for instance, *Egr1*, *Ndrg1*, *Hilpda* and *Atf3*, were significantly elevated in TBHP-induced chondrocytes, whereas these ferroptotic drivers were evidently declined by transfection with *Gfi1*-overexpression plasmid (Fig. 2C and D). In addition, GO and GSEA analysis were adopted to evaluate the functional alternation. We observed the upregulated expression of genes enriched in GO items of reactive oxygen species metabolic process, divalent metal ion transport, cell response to lipid, cellular response to metal ion and cellular response to reactive oxygen species between TBHP vs Con groups (Fig. 2E). Meanwhile, the downregulated expression of genes enriched in GO items of cellular response to metal ion and positive regulation of ion transport were shown in TBHP + *Gfi1*-OE vs TBHP groups (Fig. 2F). Furthermore, GSEA analysis indicated that GO items of positive regulation of cellular response to oxidative stress, positive regulation of oxidative stress induced cell death, positive regulation of response to oxidative stress and regulation of reactive oxygen species biosynthetic process, were less enriched in TBHP + *Gfi1*-OE group than TBHP group (Fig. 2G). These RNA sequencing data indicate the anti-ferroptotic potential of Gfi1 in chondrocytes.

### *3.3* *Gfi1* knockdown aggravates chondrocyte damage by sensitizing to ferroptosis

**Fig. 3:**
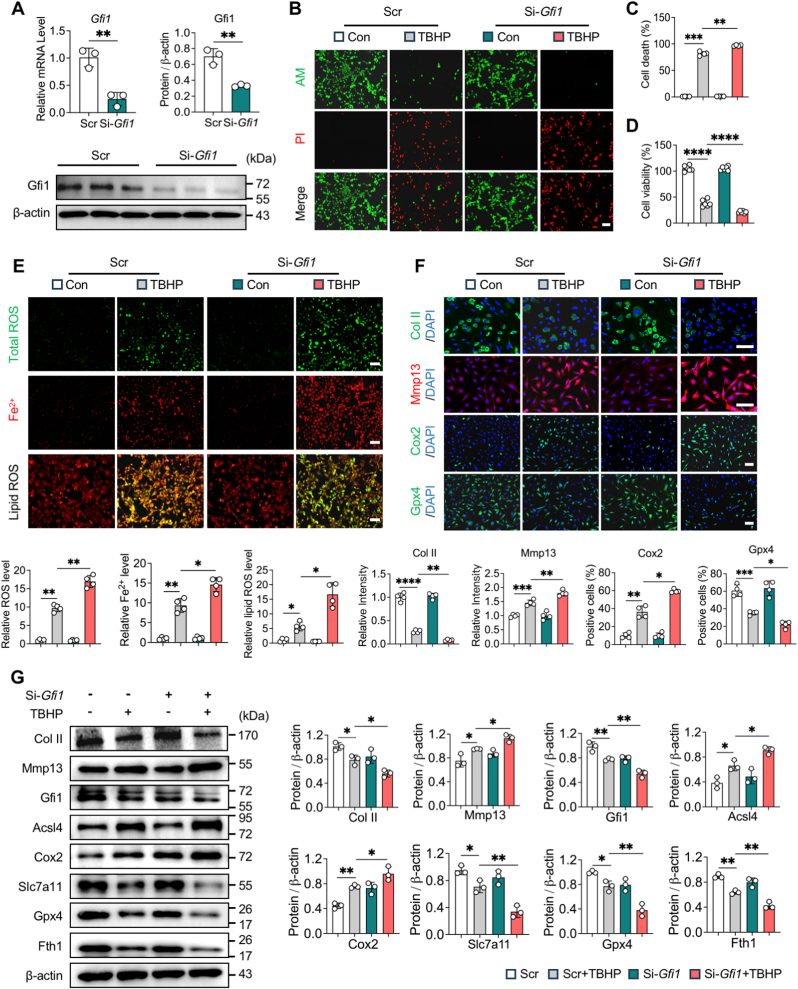
***Gfi1* knockdown aggravates chondrocyte damage by sensitizing to ferroptosis. (A)** qPCR and western blot analysis of Gfil expression in primary mouse chondrocytes by *Gfil* siRNA treatment (n = 3). **(B, C)** AM/PI staining **(B)** and quantitative analysis **(C)** of primary mouse chondrocytes induced by 100 μM TBHP for 5 h with or without pretreated *Gfi1* siRNA (n = 4). **(D)** Cell viability of primary mouse chondrocytes as treated in **(B)** (n = 6). **(E)** DCFH-DA (ROS), FerroOrange (Fe^2+^), C11-BODIPY (lipid ROS) staining and quantitative analysis of primary mouse chondrocytes as treated in **(B)** (n = 4). **(F)** Immunofluorescence (IF) staining and quantitative analysis of Col II, Mmp13, Cox2 and Gpx4 in primary mouse chondrocytes as treated in **(B)** (n = 4). **(G)** Western blot analysis and quantification of indicated proteins in primary mouse chondrocytes as treated in **(B)** (n = 3). Scale bars, 100 μm. Two-tailed unpaired t-test **(A)** or one-way ANOVA with Tukey's post-hoc test **(B-G)**. Data are presented as mean ± SD. ∗P < 0.05; ∗∗P < 0.01; ∗∗∗P < 0.001; ∗∗∗∗P < 0.0001.

To validate the impact of *Gfi1* deficiency in ferroptosis, we interfered *Gfi1* expression in chondrocytes and subsequently evaluated the alteration of ferroptosis and cartilage metabolic markers. Firstly, we successfully knocked down the expression of *Gfi1* in chondrocytes by siRNA both at mRNA and protein levels (Fig. 3A). AM/PI staining and CCK8 assay demonstrated that *Gfi1* knockdown significantly increased the ratio of PI ^+^ chondrocytes (dead cells) and reduced cell viability under TBHP stimulation (Fig. 3B–D). Moreover, the levels of representative ferroptotic markers, including total ROS, lipid ROS and Fe^2+^ accumulation, were significantly raised by *Gfi1* knockdown (Fig. 3E). IF staining showed that the relative IF intensity of Mmp13 and percentage of Cox2^+^ cells were markedly upregulated while the relative IF intensity of Col II and percentage of Gpx4^+^ cells were substantially descended in TBHP-induced chondrocytes after *Gfi1* knockdown, indicating *Gfi1* knockdown aggravated ferroptosis and imbalance of chondrocytes’ ECM anabolism-catabolism (Fig. 3F). Furthermore, western blot and quantification verified the detrimental effect of *Gfi1* knockdown in ferroptotic chondrocytes, as evidenced by elevated levels of cartilage catabolic marker (Mmp13) and ferroptotic driver (Acsl4, Cox2) and descended levels of cartilage anabolic marker (Col II) and ferroptotic suppressors (Slc7a11, Gpx4, Fth1) (Fig. 3G). These results suggest that *Gfi1* knockdown makes chondrocytes more sensitive to ferroptosis and exacerbates chondrocyte damage.

### *3.4* *Gfi1* overexpression protects chondrocytes from ferroptosis

**Fig. 4:**
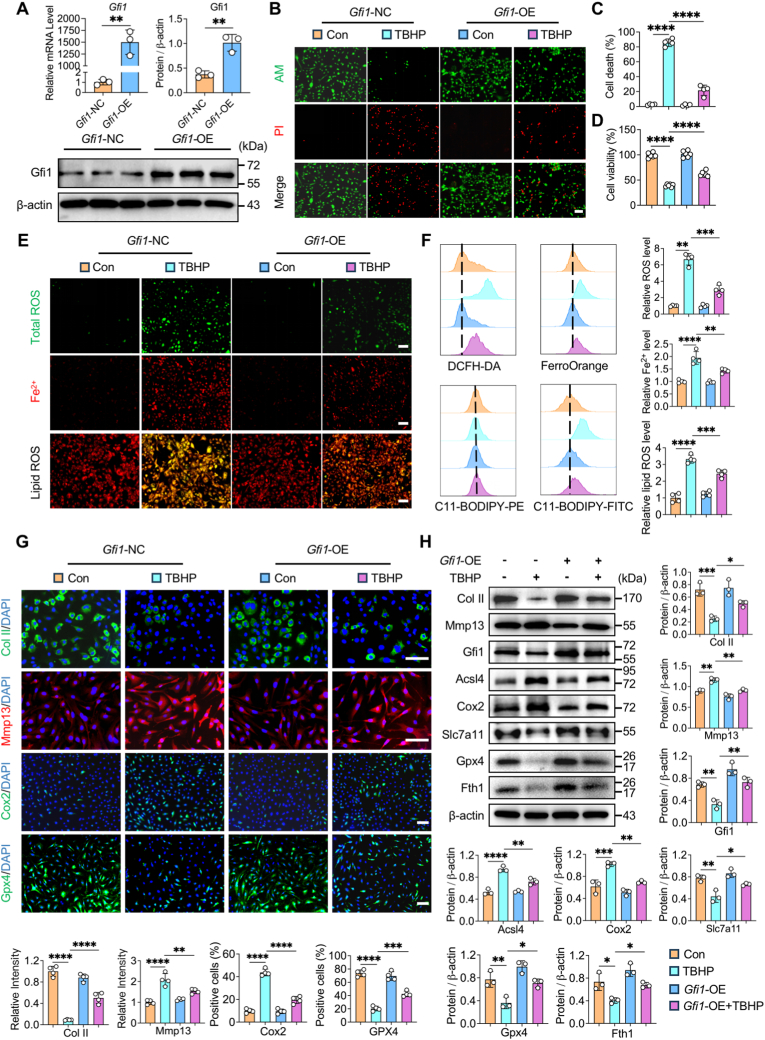
***Gfi1* overexpression protects chondrocytes from ferroptosis. (A)** qPCR and western blot analysis of Gfil expression in primary mouse chondrocytes by *Gfi1-*overexpression plasmid treatment (n = 3). **(B, C)** AM/PI staining **(B)** and quantitative analysis **(C)** of primary mouse chondrocytes induced by 100 μM TBHP for 5 h with or without pretreated *Gfi1-*overexpression plasmid (n = 4). **(D)** Cell viability of primary mouse chondrocytes as treated in **(B)** (n = 6). **(E, F)** DCFH-DA (ROS), FerroOrange (Fe^2+^), C11-BODIPY (lipid ROS) staining **(E)** and related flow cytometry analysis and quantification **(F)** of primary mouse chondrocytes as treated in **(B)** (n = 4). **(G)** Immunofluorescence (IF) staining and quantitative analysis of Col II, Mmp13, Cox2 and Gpx4 in primary mouse chondrocytes as treated in **(B)** (n = 4). **(H)** Western blot analysis and quantification of indicated proteins in primary mouse chondrocytes as treated in **(B)** (n = 3). Scale bars, 100 μm. Two-tailed unpaired t-test **(A)** or one-way ANOVA with Tukey's post-hoc test **(B-H)**. Data are presented as mean ± SD. ∗P < 0.05; ∗∗P < 0.01; ∗∗∗P < 0.001; ∗∗∗∗P < 0.0001.

To gain deep insight into the protective effect of Gfi1 in chondrocyte ferroptosis, we investigated the association between *Gfi1* overexpression and the alteration of ferroptosis. Firstly, the efficacy of *Gfi1* overexpression was confirmed both at mRNA and protein levels by qPCR and western blot analysis (Fig. 4A). AM/PI staining exhibited that the proportion of PI^+^ chondrocytes (dead cells) significantly reduced in *Gfi1*-overexpressed chondrocytes induced by TBHP, while cell viability was significantly elevated (Fig. 4B–D). Moreover, the analysis of ferroptotic hallmarks, including total ROS, lipid ROS and Fe^2+^ accumulation, demonstrated that *Gfi1* overexpression efficiently suppressed chondrocyte ferroptosis (Fig. 4E and F). Furthermore, western blot validated the anti-ferroptotic role of Gfi1 in protecting chondrocytes, as evidenced by decreased cartilage catabolic marker (Mmp13) and ferroptotic driver (Acsl4, Cox2) and restored cartilage anabolic marker (Col II) and ferroptotic suppressors (Slc7a11, Gpx4, Fth1) (Fig. 4H). Consistently, IF staining of treated chondrocytes manifested the similar tendency (Fig. 4G). Overall, the above data verify the protective role of Gfi1 as an anti-ferroptotic target in chondrocytes.

### *3.5* *Gfi1* overexpression alleviates cartilage degeneration by resisting ferroptosis in OA mice

**Fig. 5:**
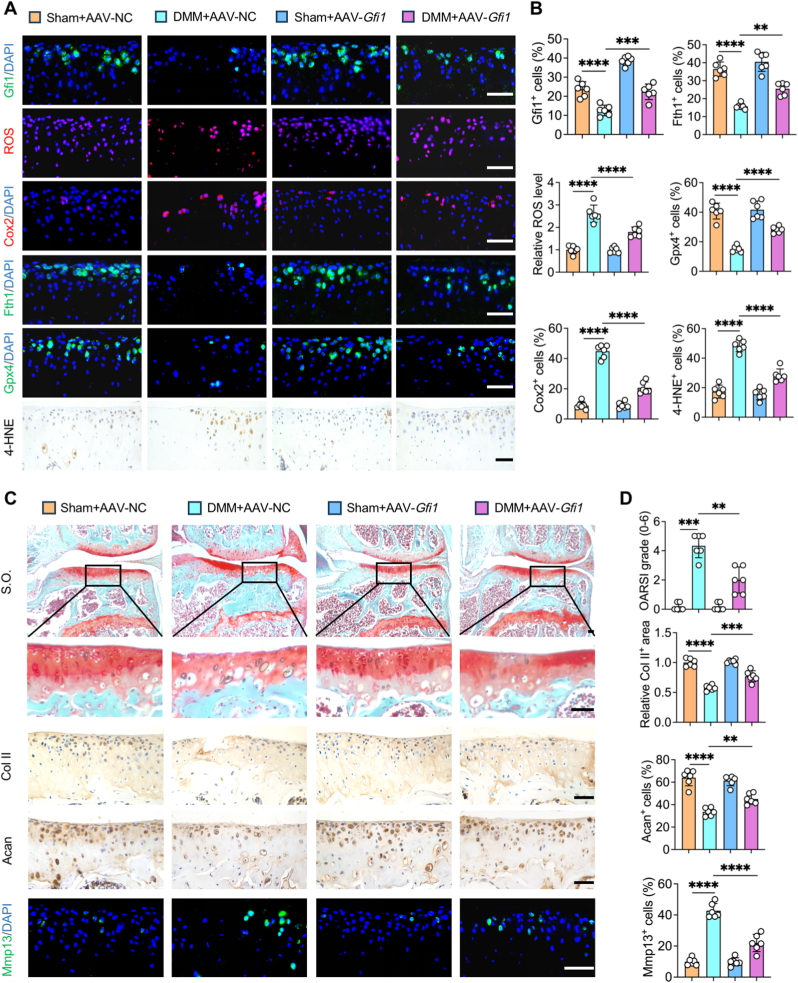
***Gfi1* overexpression alleviates cartilage degeneration by resisting ferroptosis in OA mice. (A, B)** ROS, immunohistochemical (IHC), immunofluorescence (IF) staining **(A)** and their quantitative analysis **(B)** of ROS, Gfi1, Cox2, Fth1, Gpx4, 4-HNE (n = 6). **(C, D)** Safranin O-Fast Green (S.O.) staining, IHC staining of Col II and Acan, IF staining of Mmp13 **(C)** and thier quantitative analysis **(D)** (n = 6). Scale bars, 50 μm. One-way ANOVA with Tukey's post-hoc test. Data are presented as mean ± SD. ∗∗P < 0.01; ∗∗∗P < 0.001; ∗∗∗∗P < 0.0001.

To further figure out whether *Gfi1* overexpression could alleviate cartilage degeneration by resisting ferroptosis in vivo, we performed intra-articular injection of AAV-*Gfi1* to overexpress *Gfi1* in the articular cartilages. The increased proportion of Gfi1^+^ chondrocytes in AAV-*Gfi1* group verified the efficiency of *Gfi1* overexpression in the cartilages (Fig. 5A and B). Noticeably, histological examinations also exhibited that the ROS level and percentage of Cox2^+^ and 4-HNE^+^ cells were significantly declined while the percentage of Fth1^+^ and Gpx4^+^ cells were evidently elevated in *Gfi1*-overexpressed OA cartilages (Fig. 5A and B). These results indicated the anti-ferroptotic effect of Gfi1 in vivo. Then, the assessment of the protective effects of Gfi1 on OA cartilages were conducted. Our results illustrated that intra-articular injection of AAV-*Gfi1* manifested evident cartilage-protective role in OA model, according to S.O. staining and relevant OARSI score (Fig. 5C and D). In addition, compared to DMM + AAV-NC group, DMM + AAV-*Gfi1* group promoted the restoration of ECM metabolism homeostasis in cartilages, as demonstrated by increased Col II^+^ areas and Acan^+^ chondrocytes and reduced Mmp13^+^ chondrocytes (Fig. 5C and D). Taken together, Gfi1 is essential for alleviating OA cartilage degeneration by resisting ferroptosis.

### *3.6* *Gfi1* overexpression ameliorates OA progression

**Fig. 6:**
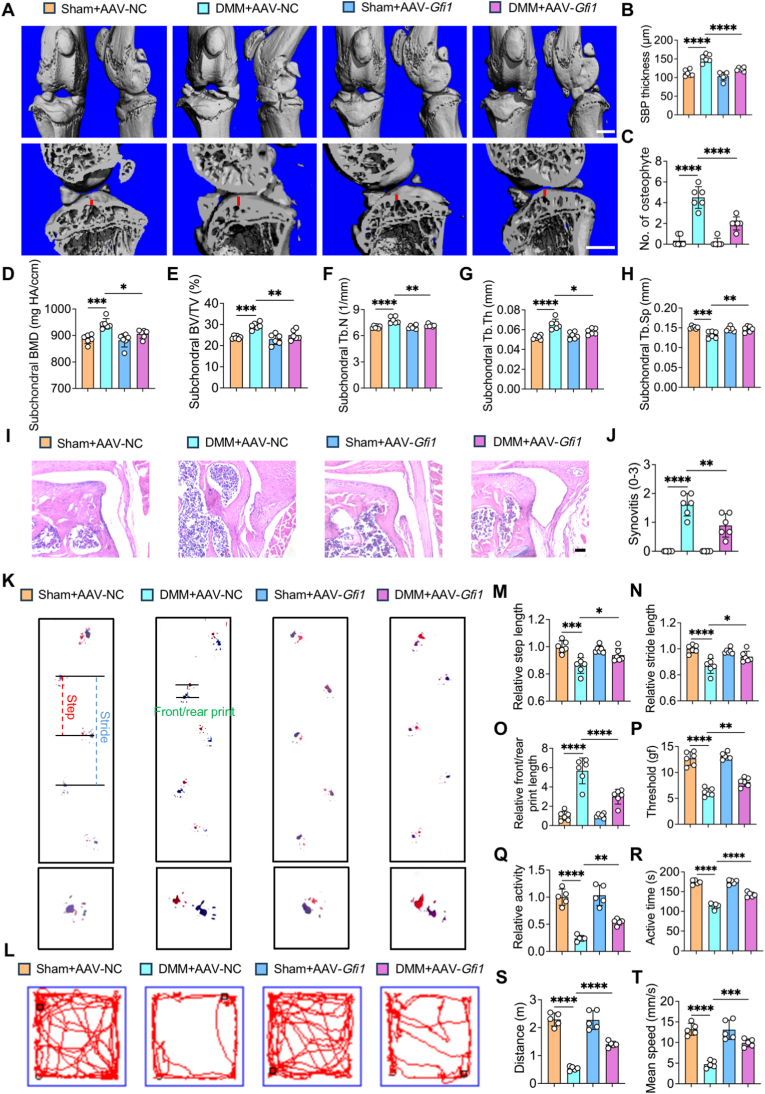
***Gfi1* overexpression ameliorates OA progression. (A**–**C)** Three-dimensional (3D) reconstructed images of mice knee joints, the sagittal view of the medial joint compartment **(A)** and quantification of the thickness of subchondral bone plate (SBP) **(B)** and osteophyte number **(C)** in mice knee (n = 6). Red line indicates the thickness of SBP. **(D**–**H)** Micro-CT analysis of subchondral bone mineral density (BMD) **(D)**, the ratio of bone volume to tissue volume (BV/TV) **(E)**, trabecular number (Tb.N) **(F)**, trabecular thickness (Tb.Th) **(G)** and trabecular separation (Tb.Sp) **(H)** in mice knee (n = 6). **(I, J)** Hematoxylin & eosin (HE) staining **(I)** and quantitative analysis (synovitis grade) **(J)** of mice knee (n = 6). **(K, M-O)** Representative mice footprint images **(K)** and gait analysis **(M**–**O)** (n = 6). Red dot line represents step length. Blue dot line represents stride length. Green dot line represents front/rear print length. **(P)** Pain sensitivity of mice which is measured by paw withdrawal mechanical threshold via von Frey test (n = 6). **(L, Q-T)** Representative track plots of mice spontaneous activity in open field tests **(L)** and quantitative analysis of relative activity **(Q)**, active time **(R)**, distance **(S)** and mean speed **(T)** during the 3 min experimental period (n = 6). Scale bars, 1 mm **(A)** and 50 μm **(I)**. One-way ANOVA with Tukey's post-hoc test. Data are presented as mean ± SD. ∗P < 0.05; ∗∗P < 0.01; ∗∗∗P < 0.001; ∗∗∗∗P < 0.0001.

Given that OA is recognized as a whole-joint disease involving cartilage, subchondral bone and synovium [27], we also performed general assessment on the severity of osteophyte formation, subchondral bone sclerosis and synovitis in OA. We utilized micro-CT analysis and 3D reconstruction to evaluate the bone state of mice knee joints, and discovered that aggravated osteophyte formation and subchondral bone sclerosis in DMM surgery-induced mice were markedly mitigated after AAV-*Gfi1* treatment (Fig. 6A–H). In detail, the number of osteophytes and the parameters of subchondral bone (SBP, BMD, BV/TV, Tb.N, Tb.Th) were declined, while Tb.Sp was upregulated in *Gfi1*-overexpressed mice OA joints (Fig. 6A–H). Moreover, in contrast to the DMM + AAV-NC group, synovitis was significantly improved and quantified with lower synovitis scores after the intra-articular injection of AAV-*Gfi1* (Fig. 6I and J).

Furthermore, in order to assess the functional alteration of mice knee, we performed a series of experiments to explore the behavior performance of OA mice with or without intra-articular injection of AAV-*Gfi1.* Firstly, we performed the gait analysis with their hind paws dipped with red ink and front paws dipped with blue ink to monitor their moving footprints. We found that mice in DMM + AAV-*Gfi1* group exhibited better moving gait than DMM + AAV-NC group, as evidenced by increased length of step and stride and reduced front/rear print length (Fig. 6K, M to O). In contrast to DMM + AAV-NC group, von Frey test showed the higher threshold of mice paw withdrawal, indicating the lower pain sensitivity in DMM + AAV-*Gfi1* group (Fig. 6P). Furthermore, mice spontaneous moving trajectory was recorded in a 50 cm × 50 cm open field for 3 min by a tracking system [28,29]. The quantification results indicated that DMM surgery-induced mice possessed poorer motor capacity than Sham group while this discrepancy was reversed by AAV-*Gfi1* treatment, as evidenced by remarkably improved motor performance including increased activity, extended active time, augmented distance and accelerated mean speed (Fig. 6L, Q to T). In summary, *Gfi1* overexpression ameliorates OA progression via reducing subchondral bone sclerosis and osteophyte formation, alleviating synovitis and improving behavior performance.

### 3.7 Gfi1 suppresses chondrocyte ferroptosis by inactivating MAPK signaling pathway

**Fig. 7:**
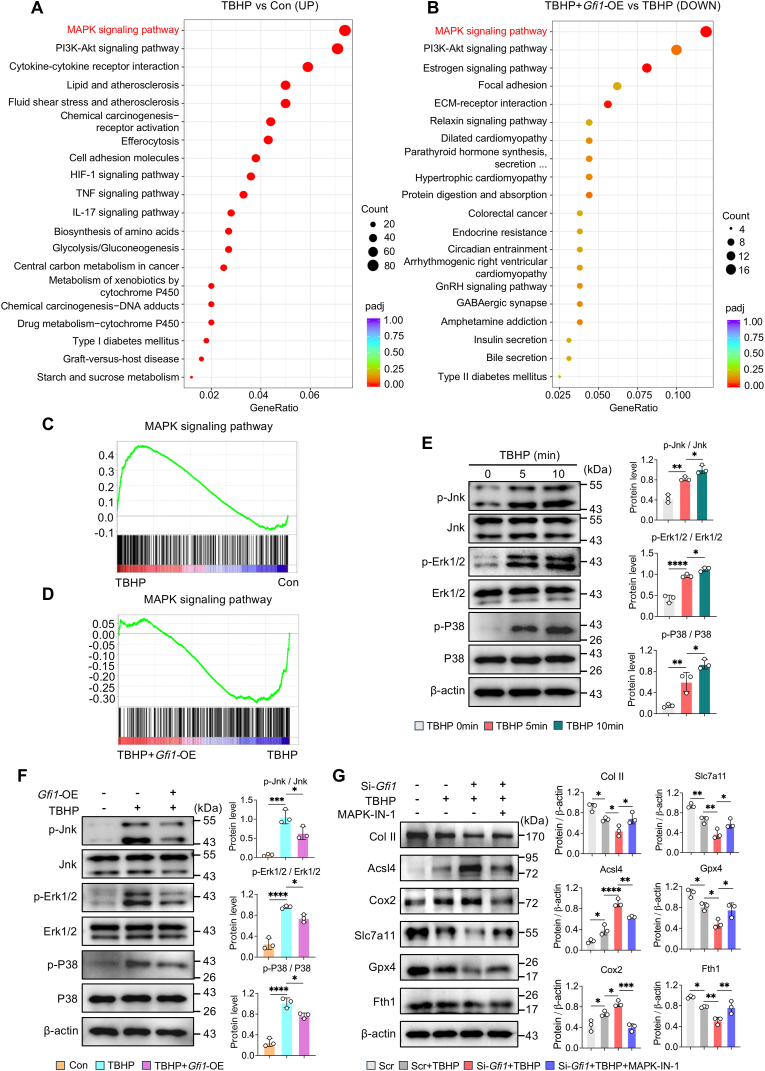
**Gfi1 suppresses chondrocyte ferroptosis by inactivating MAPK signaling pathway. (A, B)** Kyoto Encyclopedia of Genes and Genomes (KEGG) analysis of the significantly upregulated genes between TBHP vs Con groups **(A)** and downregulated genes between TBHP + *Gfi1*-OE vs TBHP groups **(B)**. **(C, D)** Gene Set Enrichment Analysis (GSEA) in RNA sequencing. **(E)** Western blot analysis and quantification of MAPK signaling pathway-related proteins in primary mouse chondrocytes induced by 100 μM TBHP for 5min or 10min (n = 3). **(F)** Western blot analysis and quantification of MAPK signaling pathway-related proteins in primary mouse chondrocytes induced by 100 μM TBHP for 10min with or without pretreated *Gfi1-*overexpression plasmid (n = 3). **(G)** Western blot analysis and quantification of indicated proteins in primary mouse chondrocytes induced by 100 μM TBHP for 5h with or without *Gfi1* siRNA or 10 μM MAPK-IN-1 treatment (n = 3). One-way ANOVA with Tukey's post-hoc test. Data are presented as mean ± SD. ∗P < 0.05; ∗∗P < 0.01; ∗∗∗P < 0.001; ∗∗∗∗P < 0.0001.

To explore the molecular mechanisms through which *Gfi1* overexpression suppressed chondrocyte ferroptosis, we further analyzed preceding RNA sequencing data. KEGG analysis demonstrated that MAPK signaling pathway exhibited the most remarkable variation, which was highly enhanced in TBHP vs Con groups and evidently weakened in TBHP + *Gfi1*-OE vs TBHP groups (Fig. 7A and B). GSEA analysis further showed that MAPK signaling pathway was more enriched in TBHP group than Con group while less enriched in TBHP + *Gfi1*-OE group than TBHP group (Fig. 7C and D). Given that MAPK signaling pathway has been reported to participate in ferroptosis [30], we speculate that Gfi1 might suppress chondrocyte ferroptosis by inactivating MAPK signaling pathway. Western blot and quantitative analysis confirmed this hypothesis, as verified by the elevated phosphorylation levels of MAPK (p-Jnk, p-Erk1/2, p-P38) in TBHP-induced chondrocytes, whereas restrained by pretreated *Gfi1*-overexpression plasmid (Fig. 7E and F). Moreover, the application of MAPK-IN-1, a MAPK signaling pathway inhibitor in *Gfil*-silenced chondrocytes could help to resist ferroptosis, as evidenced by descended levels of ferroptotic drivers (Acsl4, Cox2) and raised levels of ferroptotic suppressors (Slc7a11, Gpx4, Fth1) and restore chondrocytes’ ECM homeostasis by upregulating the expression of Col II (Fig. 7G). According to these results, we identify MAPK signaling pathway as the key downstream mechanism, against which Gfi1 exerts its anti-ferroptotic effect in OA chondrocytes.

## 4 Discussion

Increasing investigations have revealed the involvement of chondrocyte ferroptosis in OA evolution [[10], [11], [12]]. However, there still exist limited treatments targeting at the pathological characteristic of chondrocyte ferroptosis for alleviating OA deterioration. This study verifies Gfi1 as a promising anti-ferroptotic target in OA chondrocytes, as demonstrated by resistant capability to ferroptosis in TBHP-induced primary mouse chondrocytes and DMM surgery-induced mice OA cartilages. Mechanistically, MAPK signaling pathway is bioinformatically screened and biologically validated as the downstream modulator of Gfi1 exerting anti-ferroptotic role in OA chondrocytes.

Gfi1 is conventionally known as a hematopoietic transcription factor, which plays a dispensable role in cell survival during normal or aberrant hematopoiesis process. As previously shown, Gfi1 could protect hematopoietic stem cells and multiple myeloma cells via suppressing cell death-related genes, conversely, Gfi1 deficiency contributed to increased cell death [31,32]. Additionally, Gfi1's salvaging impact on cell survival was also unveiled in other fields, such as cardiomyopathy and otopathy [33,34]. These studies implied the anti-cell death potential of Gfi1, but the role of Gfi1 in OA has not been investigated. Distinctively, this study firstly probed into the effect of Gfi1 in OA chondrocyte ferroptosis. We found the expression of Gfi1 was declined in ferroptotic chondrocytes both in vitro and vivo. Moreover, negative correlation of Gfi1 with ferroptosis was illuminated via RNA sequencing analysis and dually verified by *Gfi1* knockdown or overexpression experiments.

Similar to previous studies [11,25,26], we selected TBHP to simulate OA ferroptotic environment. Application of representative ferroptosis-related probes (DCFH-DA, FerroOrange, C11-BODIPY) verified the successful establishment of in vitro chondrocyte ferroptosis model, as evidenced by higher expression levels of ferroptotic hallmarks (ROS, Fe^2+^, lipid peroxidation) after TBHP treatment. Moreover, to exclude the interference from TBHP-induced other modes of cell death, we verified the rescue effect of ferroptosis inhibitor (Fer-1) on chondrocyte death, while neither the apoptosis inhibitor (ZVAD) nor necroptosis inhibitor (Necro) exerted obvious action (Supplementary Fig. 1A–C). Based on this model, we explored the underlying mechanism of Gfi1 resisting to chondrocyte ferroptosis via RNA sequencing and observed the most significant alteration of MAPK signaling pathway in KEGG analysis. As a family member of serine/threonine protein kinases, mitogen-activated protein kinases (MAPKs) mainly consist of a series of signaling molecules including extracellular signal-regulated kinase (ERK), c-Jun N-terminal kinase (JNK) and p38 mitogen-activated protein kinase (p38 MAPK), which can be activated to play a vital role in cell proliferation, differentiation and death under various stimulus [[35], [36], [37]]. Similar to previous studies [35,38], we also observed the evident activation of MAPK signaling pathway in ferroptotic chondrocytes whereas remarkably repressed by *Gfi1* overexpression, as demonstrated by less GSEA enrichment and downregulated phosphorylation levels of MAPK signaling pathway-related proteins (p-Jnk, p-Erk1/2, p-P38). Thus, in view of these results, we reveal MAPK signaling pathway as the key downstream mediator of Gfi1 exerting anti-ferroptotic effect in OA chondrocytes. Of note, investigations in other fields, like cancer and myocardiopathy, also confirmed the pro-ferroptotic role of MAPK signaling pathway [39,40]. Gfi1-MAPK signaling axis may represent a new direction for investigating the pathological mechanism of these ferroptosis-related diseases.

In this study, we preliminarily evaluated the whole joint-protective effects of intra-articular injection of AAV-*Gfi1* in mice OA model from multiple perspectives, including cartilage degeneration, subchondral bone sclerosis, osteophyte formation, synovitis and behavior performance. This means an initial step towards clinical transformation. Nevertheless, we have to acknowledge the limitation that we didn't use a chondrocyte-specific knockout of *Gfi1* model to better validate these findings. To exclude potential confounders, IF staining of joint tissues were additionally conducted and showed no significant alterations of Gfi1 expression in intra-articular multiple tissues, such as subchondral bone and synovium (Supplementary Fig. 2), implying the relatively specific manipulation of AAV-*Gfi1* transfection on chondrocytes. Besides, to better optimize OA therapy, more agonists-targeting Gfi1 are warranted to be developed and various animal models or clinical trials are required for the assessment of safety and efficacy.

## 5 Conclusion

In summary, we observe the occurrence of Gfi1 downregulation in OA and reveal that *Gfi1* overexpression could ameliorate OA development by inhibiting chondrocyte ferroptosis via inactivation of MAPK signaling pathway. This study identifies Gfi1 as a novel therapeutic anti-ferroptotic target for cartilage degeneration, providing more clues for optimizing OA treatment strategies in clinical practice.

## Declaration of competing interest

All authors involved in this work have declared that they have no conflicts of interest.

## References

[1] Yao Q., Wu X., Tao C., Gong W., Chen M., Qu M. (2023). Osteoarthritis: pathogenic signaling pathways and therapeutic targets. Signal Transduct Targeted Ther.

[2] Hunter D.J., Bierma-Zeinstra S. (2019). Osteoarthritis Lancet.

[3] Courties A., Kouki I., Soliman N., Mathieu S., Sellam J. (2024). Osteoarthritis year in review 2024: epidemiology and therapy. Osteoarthr Cartil.

[4] Zhu R., Wang Y., Ouyang Z., Hao W., Zhou F., Lin Y. (2023). Targeting regulated chondrocyte death in osteoarthritis therapy. Biochem Pharmacol.

[5] Rim Y.A., Nam Y., Ju J.H. (2020). The role of chondrocyte hypertrophy and senescence in osteoarthritis initiation and progression. Int J Mol Sci.

[6] Tang D., Chen X., Kang R., Kroemer G. (2021). Ferroptosis: molecular mechanisms and health implications. Cell Res.

[7] Dixon S.J., Lemberg K.M., Lamprecht M.R., Skouta R., Zaitsev E.M., Gleason C.E. (2012). Ferroptosis: an iron-dependent form of nonapoptotic cell death. Cell.

[8] Chen B., Wang L., Xie D., Wang Y. (2024). Exploration and breakthrough in the mode of chondrocyte death-A potential new mechanism for osteoarthritis. Biomed Pharmacother.

[9] Xie Y., Hou W., Song X., Yu Y., Huang J., Sun X. (2016). Ferroptosis: process and function. Cell Death Differ.

[10] Sun K., Hou L., Guo Z., Wang G., Guo J., Xu J. (2023). JNK-JUN-NCOA4 axis contributes to chondrocyte ferroptosis and aggravates osteoarthritis via ferritinophagy. Free Radic Biol Med.

[11] Lv Z., Han J., Li J., Guo H., Fei Y., Sun Z. (2022). Single cell RNA-seq analysis identifies ferroptotic chondrocyte cluster and reveals TRPV1 as an anti-ferroptotic target in osteoarthritis. EBioMedicine.

[12] Miao Y., Chen Y., Xue F., Liu K., Zhu B., Gao J. (2022). Contribution of ferroptosis and GPX4's dual functions to osteoarthritis progression. EBioMedicine.

[13] Möröy T., Khandanpour C. (2019). Role of GFI1 in epigenetic regulation of MDS and AML pathogenesis: mechanisms and therapeutic implications. Front Oncol.

[14] Huang Y., Ruan R., Fang Y., Wu K., Yao L., Zhang R. (2021). GFI1 promotes the proliferation and migration of esophageal squamous cell carcinoma cells through the inhibition of SOCS1 expression. Int J Mol Med.

[15] Lee M.C., Kuo Y.Y., Chou W.C., Hou H.A., Hsiao M., Tien H.F. (2014). Gfi-1 is the transcriptional repressor of SOCS1 in acute myeloid leukemia cells. J Leukoc Biol.

[16] Khandanpour C., Möröy T. (2013). Growth factor independence 1 (Gfi1) as a regulator of p53 activity and a new therapeutical target for ALL. Oncotarget.

[17] Khandanpour C., Phelan J.D., Vassen L., Schütte J., Chen R., Horman S.R. (2013). Growth factor independence 1 antagonizes a p53-induced DNA damage response pathway in lymphoblastic leukemia. Cancer Cell.

[18] Liu Y., Gu W. (2022). p53 in ferroptosis regulation: the new weapon for the old guardian. Cell Death Differ.

[19] Wang Y., Pang X., Liu Y., Mu G., Wang Q. (2023). SOCS1 acts as a ferroptosis driver to inhibit the progression and chemotherapy resistance of triple-negative breast cancer. Carcinogenesis.

[20] Hu Z.W., Wen Y.H., Ma R.Q., Chen L., Zeng X.L., Wen W.P. (2021). Ferroptosis driver SOCS1 and suppressor FTH1 independently correlate with M1 and M2 macrophage infiltration in head and neck squamous cell carcinoma. Front Cell Dev Biol.

[21] Zheng L., Zhang C., Liao L., Hai Z., Luo X., Xiao H. (2025). Knockdown of Gfi1 increases BMSCs exosomal miR-150-3p to inhibit osteoblast ferroptosis in steroid-induced osteonecrosis of the femoral head through BTRC/Nrf2 axis. Endocr J.

[22] Gosset M., Berenbaum F., Thirion S., Jacques C. (2008). Primary culture and phenotyping of murine chondrocytes. Nat Protoc.

[23] Li J., Sun Z., Lv Z., Jiang H., Liu A., Wang M. (2021). Microtubule stabilization enhances the chondrogenesis of synovial mesenchymal stem cells. Front Cell Dev Biol.

[24] Choi W.S., Lee G., Song W.H., Koh J.T., Yang J., Kwak J.S. (2019). The CH25H-CYP7B1-RORα axis of cholesterol metabolism regulates osteoarthritis. Nature.

[25] Sun W., Lv Z., Li W., Lu J., Xie Y., Wang P. (2024). XJB-5-131 protects chondrocytes from ferroptosis to alleviate osteoarthritis progression via restoring Pebp1 expression. J Orthop Translat.

[26] Xie Y., Lv Z., Li W., Lin J., Sun W., Guo H. (2025). JP4-039 protects chondrocytes from ferroptosis to attenuate osteoarthritis progression by promoting Pink1/Parkin-dependent mitophagy. J Orthop Trans.

[27] Chen D. (2022). Osteoarthritis: a complicated joint disease requiring extensive studies with multiple approaches. J Orthop Translat.

[28] Lv Z., Wang P., Li W., Xie Y., Sun W., Jin X. (2024). Bifunctional TRPV1 targeted magnetothermal switch to attenuate osteoarthritis progression. Research.

[29] Li W., Lv Z., Wang P., Xie Y., Sun W., Guo H. (2024). Near infrared responsive gold nanorods attenuate osteoarthritis progression by targeting TRPV1. Adv Sci (Weinh).

[30] Chen Y., Fang Z.M., Yi X., Wei X., Jiang D.S. (2023). The interaction between ferroptosis and inflammatory signaling pathways. Cell Death Dis.

[31] Petrusca D.N., Toscani D., Wang F.M., Park C., Crean C.D., Anderson J.L. (2018). Growth factor independence 1 expression in myeloma cells enhances their growth, survival, and osteoclastogenesis. J Hematol Oncol.

[32] Khandanpour C., Kosan C., Gaudreau M.C., Dührsen U., Hébert J., Zeng H. (2011). Growth factor independence 1 protects hematopoietic stem cells against apoptosis but also prevents the development of a myeloproliferative-like disease. Stem Cell.

[33] Wallis D., Hamblen M., Zhou Y., Venken K.J., Schumacher A., Grimes H.L. (2003). The zinc finger transcription factor Gfi1, implicated in lymphomagenesis, is required for inner ear hair cell differentiation and survival. Development.

[34] Zheng Y., Li S., Hu R., Cheng F., Zhang L. (2020). GFI-1 protects against lipopolysaccharide-induced inflammatory responses and apoptosis by inhibition of the NF-κB/TNF-α pathway in H9c2 cells. Inflammation.

[35] Zhao C., Sun G., Li Y., Kong K., Li X., Kan T. (2023). Forkhead box O3 attenuates osteoarthritis by suppressing ferroptosis through inactivation of NF-κB/MAPK signaling. J Orthop Translat.

[36] Housmans B.A.C., Neefjes M., Surtel D.A.M., Vitík M., Cremers A., van Rhijn L.W. (2022). Synovial fluid from end-stage osteoarthritis induces proliferation and fibrosis of articular chondrocytes via MAPK and RhoGTPase signaling. Osteoarthr Cartil.

[37] Chen Z., Yue S.X., Zhou G., Greenfield E.M., Murakami S. (2015). ERK1 and ERK2 regulate chondrocyte terminal differentiation during endochondral bone formation. J Bone Miner Res.

[38] Cui T., Lan Y., Yu F., Lin S., Qiu J. (2023). Plumbagin alleviates temporomandibular joint osteoarthritis progression by inhibiting chondrocyte ferroptosis via the MAPK signaling pathways. Aging (Albany NY).

[39] Ye F., Chai W., Xie M., Yang M., Yu Y., Cao L. (2019). HMGB1 regulates erastin-induced ferroptosis via RAS-JNK/p38 signaling in HL-60/NRASQ61L cells. Am J Cancer Res.

[40] Chen W., Zhang Y., Wang Z., Tan M., Lin J., Qian X. (2023). Dapagliflozin alleviates myocardial ischemia/reperfusion injury by reducing ferroptosis via MAPK signaling inhibition. Front Pharmacol.

