# Supplementary material for: Growth factor independence 1 ameliorates osteoarthritis by inhibiting chondrocyte ferroptosis via inactivation of MAPK signaling pathway
Source: J Orthop Translat. 2025 Jul 29;54:101–14. doi: 10.1016/j.jot.2025.07.003 (PMC12332208; doi:10.1016/j.jot.2025.07.003)
Supplement: Multimedia component 1 [file mmc1.pdf]

Supplementary information

Supplementary Table 1. Clinical characteristics of OA patients from whom cartilage samples were utilized for histological staining.

No.	Gender	Age	Hight (cm)	Weight (kg)	BMI	Joint	Side	KL Score
01	Female	84	153	63	26.91	Knee	Right	4
02	Female	60	164	62	23.05	Knee	Right	4
03	Female	68	167	67	24.02	Knee	Right	4
04	Female	66	152	91	39.39	Knee	Right	4
05	Female	62	151	82	35.96	Knee	Right	4
06	Female	70	155	72.5	30.18	Knee	Left	4

No., Number; BMI, Body mass index; KL score, Kellgren-Lawrence score.

Supplementary Table 2. Clinical characteristics of OA patients from whom cartilage samples were utilized for western blot analysis.

No.	Gender	Age	Hight (cm)	Weight (kg)	BMI	Joint	Side	KL Score
01	Male	74	171	100	34.20	Knee	Left	4
02	Male	76	160	68	26.56	Knee	Right	4
03	Female	62	157	82.5	33.47	Knee	Left	4
04	Female	72	164	78	29.00	Knee	Right	4

No., Number; BMI, Body mass index; KL score, Kellgren-Lawrence score.

Supplementary Table 3. Primer sequences of qPCR.

Gene	Forward primer (5'→3')	Reverse primer (5'→3')
Col2a1	GGGAATGTCCTCTGCGATGAC	GAAGGGGATCTCGGGGTTG
Mmp13	CTTCTTCTTGTTGAGCTGGACTC	CTGTGGAGGTCACTGTAGACT
Gfi1	CCCTTTGCGTGCGAGATGT	CAGCGTGGATGACCTCTTGAA
Ptgs2	TGAGCAACTATTCCAAACCAGC	GCACGTAGTCTTCGATCACTATC
Fth1	CAAGTGCGCCAGAACTACCA	GCCACATCATCTCGGTCAAAA
β-Actin	GGCTGTATTCCCCTCCATCG	CCAGTTGGTAACAATGCCATGT

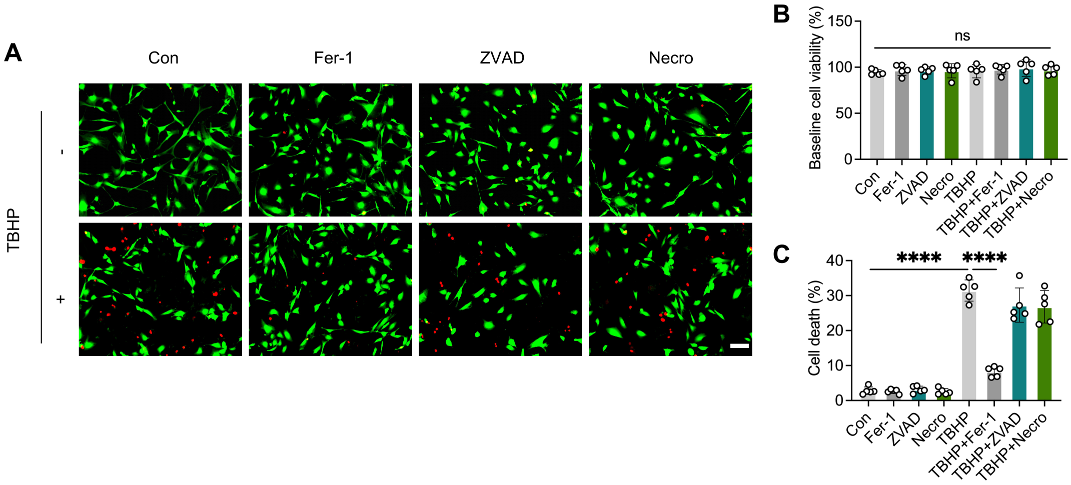

Supplementary Fig. 1. TBHP induces chondrocyte ferroptosis. (A, C) AM/PI staining **(A)** and quantitative analysis **(C)** of primary mouse chondrocytes induced by 100 μM TBHP for 5 h with or without ferroptosis inhibitor (5 μM Ferrostatin-1 (Fer-1)), apoptosis inhibitor (10 μM ZVAD-FMK (ZVAD)), or necroptosis inhibitor (10 μM Necrosulfonamide (Necro)) (n = 5). **(B)** Baseline cell viability of primary mouse chondrocytes before TBHP treatment (n = 5). Scale bars, 100 μm. One-way ANOVA with Tukey's post-hoc test. Data are presented as mean ± SD. ****P < 0.0001.

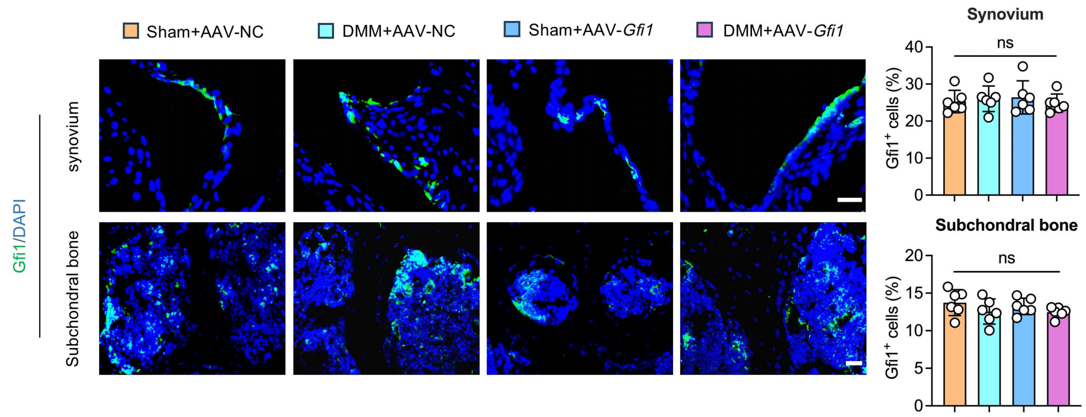

Supplementary Fig. 2. Intra-articular injection of AAV-*Gfi1* cannot overexpress *Gfi1* in synovium and subchondral bone. Immunofluorescenc (IF) staining and quantitative analysis of Gfi1 in synovium and subchondral bone (n = 6). Scale bars, 25 μm. One-way ANOVA with Tukey's post-hoc test. Data are presented as mean ± SD.
